# Continuity of care is associated with satisfaction with local health care services

**DOI:** 10.1186/s12875-020-01251-5

**Published:** 2020-09-04

**Authors:** E. Lautamatti, M. Sumanen, R. Raivio, K. J. Mattila

**Affiliations:** 1 Faculty of Medicine and Health Technology, Tampere University, Tampere and Centre for General Practice of the Pirkanmaa Hospital District, Tampere, Finland; 2 Päijät-Häme Joint Authority for Health and Wellbeing, Primary Health Care, Lahti, Finland

**Keywords:** Primary health care, Population-based, Patient satisfaction, Continuity of care, Depression, Health care services, Questionnaire study, General practice, Finland

## Abstract

**Background:**

Satisfaction is a major element in assessing quality of care. It has decreased in Finland in recent decades as well as continuity of care. We investigated which demographic, health-related, and local health care service factors, especially continuity of care, are associated with the population’s satisfaction with local health care services.

**Methods:**

The data are part of the Health and Social Support (HeSSup) study’s follow-up questionnaire in 2012. The study is based on a random Finnish population sample. Satisfaction was studied based on the question “How satisfied are you with your local health care services?” Demographic factors, obesity, self-assessed health status, depressive mood (BDI-12 questionnaire), New York Heart Association class, and chronic diseases were asked in the questionnaire. Questions describing local health care services were also presented. We assessed the association of an assigned and named GP and the respondents’ proactivity in contacting the same doctor with satisfaction. We used crosstabulation and binary logistic regression in the analyses.

**Results:**

The Health and Social Support study was answered in 2012 by 15,993 participants (45.4%) and majority (61.3%) was satisfied with their local health care services. An assigned and named GP (OR 1.79; 95% CI 1.67–1.92) and the respondent’s proactivity in contacting the same doctor (OR 1.23; 95% CI 1.15–1.32) were associated with satisfaction in the adjusted multivariate analysis. BDI score < 19 had the strongest association with satisfaction (OR 1.91; 95% CI 1.65–2.23). Older participants, males, and those in a relationship were more likely to be satisfied.

**Conclusions:**

A named GP in primary care proved to have a positive correlation with patient satisfaction. Depression was associated with decreased satisfaction. A named GP indicates continuity of care, and it should be seriously considered when planning treatment for patients with chronic conditions.

## Background

Satisfaction with health care services influences the population’s wellbeing, and measuring satisfaction is one way of gauging the quality of services. The aim of the health care system is to produce services that prevent illnesses and enhance wellbeing, treatment, and rehabilitation. Higher quality of services results in a higher satisfaction.

Health care systems can be assessed from the perspective of the organization, provider, population, or individual patient. The necessity and utility of health care services differ across the population. Opinions are affected by the previous experiences, perceptions and expectations of the patient or family. Better communication skills, friendliness, empathy, a patient-centred attitude, and shared decision-making increase patient satisfaction [1–3], as does trust in the physician [4]. The reputation and public opinion of health care services influence opinion as well, and the population’s opinion is an important factor when improving health care services. In health care systems like the one found in Finland, the population’s satisfaction with services reflects the municipalities’ capability to provide and organize the service needed and meet the citizens’ expectations.

Finland’s public health law of 1972 obliged municipalities to organize health care services. Health care centres were built in every municipality in Finland, and general practitioners played a crucial role in providing services. In the 1980s, the government and Social Insurance Institution of Finland trialled and later recommended to municipalities the system of assigning and naming a GP personally responsible for diagnosing and treating each citizen [5]. An economic depression and lack of GPs drove the system into crisis in the 1990s. Municipalities started to use alternative methods of organizing services. Instead of booking an appointment with GP, patients get triage done by nurses or teams. A personal listing or a doctor-nurse pair in treating patients became common. Few municipalities continued using named GPs, which enabled personal doctor-patient relationships. Continuity of care, which was evident with a named GP, was no longer the basis of population’s health care services.

In Finland, public services are financed by municipal taxes, national government subsidies, user charges, and national sickness insurance payments. Municipalities can produce health care services themselves or buy or outsource services to private providers, but the municipalities are still accountable for the organizational procedures. Municipalities provide wide range of health care services including child and maternity care, health promotion and care, student health care and rehabilitation services. Physicians in health care centres can consult specialists in secondary care or make a referral when necessary. Municipalities are also responsible by law to organise secondary care, which is usually bought from a third party. Occupational health has an obligation to take care of the workforce using a preventive approach, and employers frequently also organize the treatment of diseases for employees. In addition to public services, Finns can also choose to buy health care services from private practices, for which they receive compensation from National Insurance and private insurance [6]. Population forms the opinion of the health care system using all or some of the services. Characteristics of the population influence satisfaction also.

The population’s interest in health care services can be roughly divided into two: the interests of the healthy and the interests of the sick. Healthy people have only minor or short-term illnesses. They have expectations of the health care system, although they do not need it regularly. Their opinion illustrates the trust of the population towards the system.

People with chronic somatic or mental diseases can be considered chronically ill. They need and use health care services regularly. The patient’s perceptions, previous experiences, and fulfilment of expectations influence health care satisfaction [7, 8]. Relevant factors affecting patient satisfaction include the population’s demographic characteristics, health status, and chronic illnesses [7, 9–18]. Among the Finnish population, multimorbidity is rising [19]. Accessibility, the patient-provider relationship, and continuity of care are strongly associated with patient satisfaction [1, 2, 10, 20–25].

Continuity of care is a multidimensional phenomenon, and the dimensions are often defined in terms of informational, longitudinal, and interpersonal continuity. Informational continuity is the demand for information about the patient’s problems and past treatments from the patient to the health care provider or between providers. Longitudinal or chronological continuity and information transfer are needed to create a continuous patient-doctor relationship over time. Time and interpersonal continuity provide a sense of confidentiality and trust between the patient and health care provider. Managerial continuity does not demand an interpersonal relationship; rather, it can be considered as co-ordinating care while aiming at the consistent management of the patient’s changing needs [26, 27].

Satisfaction has been evaluated in the Finnish health care system via patient satisfaction surveys. This gives us a picture of the opinions of people using the services, but not of the overall population. Accessibility and continuity of care have deteriorated in recent decades, as has patient satisfaction [22]. The loss of the assigned and named GP system, the ageing population, and increasing morbidity are thought to be accountable for the change in satisfaction. Factors associated with satisfaction among the unselected population have not been investigated in Finland.

Our guiding research question was the following: Is the populations’ satisfaction with local health care associated with demographic, health status, and/or local service characteristics? Our objective is to study which independent factors (gender, age, native language, relationship, education, health status, reported chronic diseases, obesity, functional limitations, depressive mood, assigned and named GP, opportunity to contact occupational health and proactivity in contacting the same doctor) are associated with the dependent factor (satisfaction).

## Methods

**Fig. 1:**
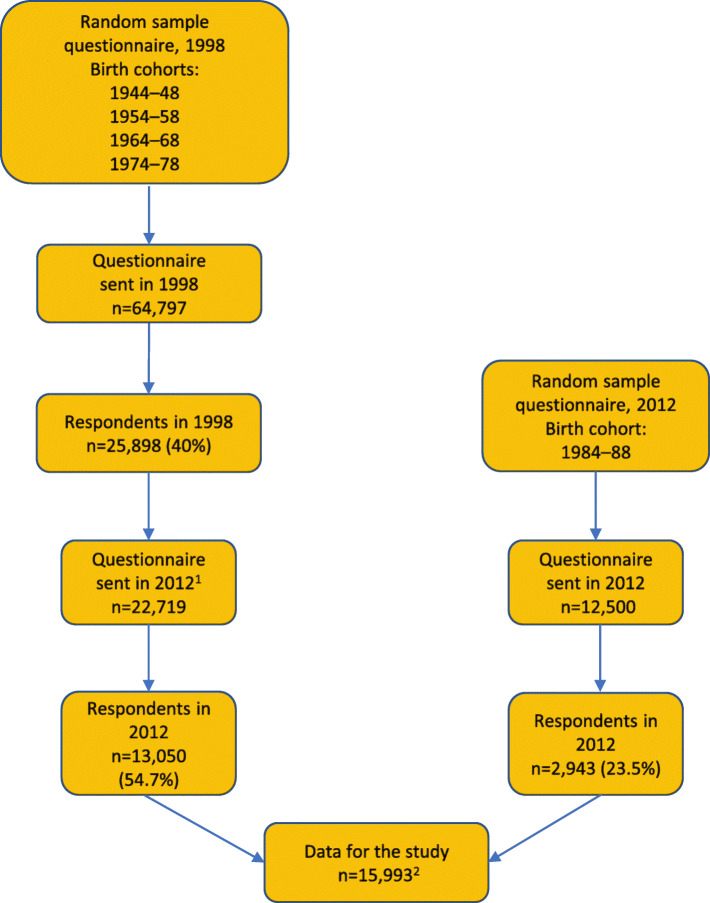
The procedure of forming the data for the study. 1) Participants who were deceased, had emigrated, or had declined delivery of their address from the Finnish Population Register were excluded. 2) 45.4% of posted questionnaires and 20.7% of the sample

The participants and information were drawn from the Health and Social Support (HeSSup) Study cohort. The first questionnaire was sent in 1998 to a random sample of 64,797 working-aged individuals drawn from the Finnish Population Register. The sample comprised four birth cohorts: 1944–1948, 1954–1958, 1964–1968, and 1974–1978. The response rate was 40% (*n* = 25,898). A second follow-up questionnaire was sent in 2012 to the respondents of the 1998 questionnaire. Those who had declined delivery of their address from the Finnish Population Register, were emigrated, or had died were excluded. The data were complemented with a random sample of the 1984–1988 birth cohort from the Finnish Population Register. The number of participants was thus raised to 15,993, and the response rate was 45.4% (Fig. 1). According to the non-response analysis in 1998, respondents and non-respondents were comparable with respect to the most important demographic variables, including gender and age distribution. Moreover, differences in physical health between the participants and the general population were minor [28, 29].

The question “How satisfied are you with your local health care services?” assessed people’s satisfaction. The options in answering were “very satisfied”, “fairly satisfied”, “fairly unsatisfied”, “very unsatisfied”, and “I can’t say/I don’t know/I’m not sure”. The first two options were classified as satisfied, while the other options do not express satisfaction and were thus classified as not satisfied.

Demographic factors were divided into two classes based on the participants’ designations. Age was cut at 65 years. Marital status was classified as in a relationship or not in a relationship. Participants in a relationship were those who reported being married, re-married, or in a common-law marriage. Patients with higher education had a degree from a college, university, or polytechnic.

Health status was categorized on a five-point Likert scale. People’s assessment of their health as good or fairly good was considered good. Other assessments were categorized as poor. The patients’ chronic diseases were based on their response. Answers to the question “Has a physician ever said that you have or have had…” included 32 options. The alternatives were “Yes” or “No”. The last of the options was an open field in which the patient could record other diseases not mentioned on the form. The participant was considered to have the disease if the answer to the specific question was “Yes”. If the answer was “No” or blank, the respondent was categorized into the “no disease reported” group. Some 26 of the 32 were categorized as chronic (Table 1). The categorization and description of chronic diseases were based on NICE guidelines [30]. Participants who had one or more chronic diseases were categorized into the “Reported chronic diseases” group (*n* = 10,273), with the others categorized into the “No reported chronic disease” group (*n* = 5720).

**Table 1:** Characteristics and satisfaction of the respondents in the HeSSup 2012 questionnaire

Characteristics	Satisfied						
	Yes		No		All	Difference	
	n	%	n	%	n	% points	*p*-value
DEMOGRAPHIC FACTORS							
Gender							
female	6149	61.0	3924	39.0	10,073		
male	3657	63.3	2121	36.7	5778	2.2	0.005
Age							
≤ 64 years	7545	59.8	5071	40.2	12,616		
≥ 65 years	2261	69.9	974	30.1	3235	10.1	< 0.001
Native language							
Finnish	8736	61.6	5443	38.4	14,179		
Swedish	1070	64.0	602	36.0	1672	2.4	0.058
Relationship							
No	2518	58.8	1762	41.2	4280		
Yes	7255	63.0	4258	37.0	11,513	4.2	< 0.001
Education							
higher	3475	59.9	2326	40.1	5801		
lower	6265	62.9	3695	37.1	9960	3.0	< 0.001
HEALTH STATUS							
Health status							
good	9285	62.0	5687	38.0	14,972		
poor	465	58.7	327	41.3	792	3.3	0.062
Reported chronic diseases							
No	3375	59.7	2283	40.3	5658		
Yes	6431	63.1	3762	36.9	10,193	3.4	< 0.001
Obesity							
BMI < 25 kg/m^2^	4605	61.4	2892	38.6	7497		
BMI ≥25 kg/m^2^	5078	62.3	3074	37.7	8152	0.9	0.265
Functional limitations							
NYHA 0–1	8918	61.8	5513	38.2	14,431		
NYHA 2–4	835	62.2	507	37.8	1342	0.4	0.097
Depressive mood							
BDI < 19	9328	62.6	5568	37.4	14,896		
BDI ≥19	376	47.2	420	52.8	796	15.4	< 0.001
REGIONAL SERVICE CHARACTERISTICS							
Assigned and named GP							
No	4743	55.6	3782	44.4	8525		
Yes	4912	70.2	2086	29.8	6998	14.6	< 0.001
Opportunity to contact occupational health							
No	3957	62.3	2392	37.7	6349		
Yes	5537	61.0	3535	39.0	9072	1.3	0.105
Proactivity in contacting the same doctor							
No	3509	56.7	2676	43.3	6185		
Yes	6208	65.2	3307	34.8	9515	8.5	< 0.001

Body Mass Index (BMI) was categorized as low BMI (BMI < 25) and high weight (BMI ≥25). Depressive mood was assessed by Beck’s Depression Inventory [31]. Values < 19 describe a mood that is normal or mildly depressive, while values ≥19 describe a depressive mood that is moderate or difficult. The New York Heart Association (NYHA) classification was used to describe the respondent’s functional limitation. The NYHA scores [32] were divided into the classes 0–1 and 2–4, with 0 meaning no symptoms of dyspnoea during physical activity (Table 1).

Local health care service characteristics were represented by questions describing continuity of care and the option of using occupational health care services. Continuity of care was evaluated by the questions “Do you have an assigned and named GP at your health care centre?” and “Do you seek to contact the same physician when you need a physician’s help or advice?” The accessibility of alternative health care services was studied by asking “Can you use occupational health services when needed for your illness, symptom, or problem?” The answer options were “Yes” and “No”.

The descriptive statistics including frequencies and percentages were used. Statistical significance was evaluated using Pearson’s chi-square test, odds ratios, and 95% confidence intervals. In the binary adjusted logistic regression analysis, the dependent factor was satisfaction with local health care services. Factors having a statistically significant association with satisfaction were included in the multivariate regression analysis. The analyses were performed using SPSS v.25.

## Results

The majority of participants (61.3%) were satisfied with their local health care services. The proportion of satisfied respondents was highest among those who had an assigned and named GP (70.2%). The difference to respondents who did not have named GP was 14.6 percentage points. Depressed patients were less likely to be satisfied. The group with a BDI score < 19 had 15.4 more percentage points of satisfied respondents compared to the scoring ≥19. The mean age of the population was 47.9 years (SD 14.6). The proportion of satisfied was 10.1 percentage points greater among participants 65 and older compared to younger respondents. Difference in share of satisfied participants between proactive and not proactive in contacting the same doctor was 8.5 percentage points, proactive were more often satisfied. There were no notable differences in the share of satisfied respondents when comparing males to females, Finnish speakers to Swedish speakers, the lower educated to the higher educated, those with a lower self-assessed health status to those with a higher health status, those with a lower NYHA score to those with a higher NYHA score, and those with reported chronic diseases to those with no reported chronic diseases (Table 1).

The most common self-reported diseases were migraine (*n* = 3276), arthrosis (*n* = 2818), depression (*n* = 2760), and hypertension (*n* = 2135). The share of satisfied respondents was highest among participants with atrial fibrillation/flutter (70.9%), transient ischemic attack (TIA) (69.5%), cataract or glaucoma (69.4%), hypertension (68.5%), diabetes (68.8%), and cancer (68.5%). The difference in satisfaction was greatest among respondents with atrial fibrillation/flutter and those without (9.4 percentage points). Satisfaction with local health care services was significantly lower among respondents reporting depression or another mental disorder compared to none (from − 3.8 to − 5.5 percentage points) (Table 2).

**Table 2:** Satisfaction among respondents reporting chronic disease (n = 10,193). Relative number of satisfied respondents for each disease compared to all others without the disease. Sorted by the largest percent point difference in satisfaction

Chronic diseases	Satisfied					
	Chronic disease		Others		Difference	
	n	%	n	%	%points	p
Atrial fibrillation/flutter	389	70.9	9417	61.5	9.4	< 0.001
Cataract or glaucoma	588	69.4	9218	61.4	8.0	< 0.001
Transient Ischemic Attack	294	69.5	9512	61.7	7.8	0.001
Hypertension	1462	68.5	8344	60.8	7.7	< 0.001
Diabetes	692	68.8	9114	61.4	7.4	< 0.001
Cancer	540	68.5	9266	61.5	7.0	< 0.001
Rheumatoid arthrosis	324	68.2	9482	61.7	6.5	0.015
Stroke	109	68.1	9697	61.8	6.3	0.101
Coeliac disease	183	67.8	9623	61.8	6.0	0.044
Myocardial infarction	186	67.1	9620	61.8	5.3	0.068
Angina pectoris	253	66.9	9553	61.7	5.2	0.040
Arthrosis	1832	65.0	7974	61.2	3.8	< 0.001
Fibromyalgia	296	65.3	9605	61.8	3.5	0.122
Kidney disease	201	65.3	9510	61.8	3.5	0.215
Other neurological disease	302	64.3	9504	61.8	2.5	0.279
Epilepsy	165	64.2	9641	61.8	2.4	0.436
Brain injury	131	63.9	9675	61.8	2.1	0.545
Other chronic disease	119	62.6	9687	61.9	0.7	0.826
Long term bronchitis/emphysema	880	62.4	8926	61.8	0.6	0.683
Asthma	840	60.6	8966	62.0	−1.4	0.296
Migraine	1978	60.4	7828	62.3	−1.7	0.049
Depression	1619	58.7	8187	62.5	−3.8	< 0.001
Other mental disorder	415	57.6	9391	62.1	−4.5	0.015
Panic disorder	673	56.8	9133	62.3	−5.5	< 0.001
Eating disorder	222	56.5	9584	62.0	−5.5	0.026
Liver disease	136	56.4	9670	61.9	−5.5	0.080

In the unadjusted logistic regression analysis of the demographic characteristics, older age (OR 1.56; 95% CI 1.44–1.70) was associated with satisfaction. The associated health status factors were a BDI score < 19 (OR 1.87; 95% CI 1.62–2.16) and the respondent having a chronic disease (OR 1.16; 95% CI 1.08–1.24). Of the characteristics of the local health care services, an assigned and named GP (OR 1.88; 95% CI 1.76–2.01) and proactivity in contacting the same health care provider (OR 1.43; 95% CI 1.34–1.53) were associated with satisfaction. No significant associations were found with native language, obesity, health status, functional limitations, or opportunity to contact occupational health (Table 3).

**Table 3:** Results of unadjusted and adjusted binary logistic regression analysis to determine the association between independent factors and satisfaction^a^

Characteristics	Unadjusted analysis		Adjusted analysis	
	OR (95% Cl)	p	OR (95% Cl)	p
DEMOGRAPHIC FACTORS				
Gender				
female	1		1	
male	1.10 (1.03–1.18)	0.005	1.11 (1.04–1.20)	0.003
Age				
≤ 64 years	1		1	
≥ 65 years	1.56 (1.44–1.70)	< 0.001	1.36 (1.24–1.49)	< 0.001
Native language				
Finnish	1			
Swedish	1.11 (0.98–1.23)	0.058		
Relationship				
No	1		1	
Yes	1.19 (1.11–1.28)	< 0.001	1.14 (1.06–1.23)	0.001
Education				
higher	1		1	
lower	1.14 (1.06–1.21)	< 0.001	1.02 (0.95–1.10)	0.571
HEALTH STATUS				
Health status				
poor	1			
good	1.15 (0.99–1.33)	0.062		
Reported chronic diseases				
No	1		1	
Yes	1.16 (1.08–1.24)	< 0.001	1.07 (0.99–1.15)	0.091
Obesity (BMI)				
< 25 kg/m^2^	1			
≥ 25 kg/m^2^	1.04 (0.97–1.11)	0.265		
NYHA classification				
0–1	1			
2–4	1.02 (0.91–1.14)	0.76		
Depressive mood (BDI)				
≥ 19	1		1	
< 19	1.87 (1.62–2.16)	< 0.001	1.91 (1.65–2.23)	< 0.001
REGIONAL SERVICE CHARACTERISTICS				
Assigned and named GP				
No	1		1	
Yes	1.88 (1.76–2.01)	< 0.001	1.79 (1.67–1.92)	< 0.001
Opportunity to contact occupational health				
No	1			
Yes	0.95 (0.89–1.01)	0.105		
Proactivity in contacting the same doctor				
No	1		1	
Yes	1.43 (1.34–1.53)	< 0.001	1.23 (1.15–1.32)	< 0.001

^a^Adjusted analysis included factors that had a statistically significant association with satisfaction

In the adjusted logistic regression analyses, we included factors having a statistically significant association with satisfaction in the univariate analysis. The strongest associations were found for a lower BDI score (OR 1.91; 95% CI 1.65–2.23), an assigned and named GP (OR 1.79; 95% CI 1.67–1.92), older age (OR 1.36; 95% CI 1.24–1.49), and respondents seeking to contact the same physician (OR 1.23; 95% CI 1.15–1.32). The associations of male gender and lower education were weaker. Reported chronic diseases did not associate statistically significantly with higher satisfaction (Table 3).

## Discussion

Having a named GP in the local health care centre was associated with satisfaction as was also the patients’ proactivity in contacting the same GP. A lower BDI score was associated with satisfaction, while a higher BDI score indicating depression showed lower satisfaction. Satisfaction with health care services was higher among respondents with diseases demanding regular controls. Older age was associated with satisfaction, but there were no significant differences found between males and females.

The response rate in 1998 was 40%. Differences between the cohort and the general Finnish population were small, thus the cohort represents the population well [29]. The response rate in 2012 was 57%, which is high even internationally for a follow-up study. Considering the demanded written consent, the length and sensitive subject matter of the questionnaire, and the ageing of the cohort, this response rate provides solid ground for a population study. The number of participants was high (15,993), giving us a solid sample of the Finnish population.

The study was based on self-reported characteristics that respondents were aware of and willing to discuss. Although not found, the risk of attenuation bias exists. It is possible that the self-reported questionnaire understates morbidity, although according to the literature self-assessed health seems to be associated with objective health status [33]. However, the received data are comparable to the Finnish population, and the results of the study can thus be generalized [28].

Determining patient satisfaction based on a single question is a major limitation of this study. The population was randomly selected, and they answered the questionnaire at home. We do not know how long had elapsed since the respondents’ previous contact with a physician, and we do not know whether the respondents answered based on their experiences in primary or secondary health care. Different aspects of satisfaction (accessibility, doctor-patient relationship, facilities, quality of care) were not evaluated in our study. However, overall satisfaction was reported, and the results can be relied on. Methods of organizing health care system are complex and vary among municipalities in Finland, which makes interpretation of multidimensional population-based satisfaction survey challenging.

Using only one question to determine continuity of care is also a limitation of the study. Raivio et al. have found an assigned and named GP to be the most important factor in increasing continuity [22]. Continuity of care could be assessed over a longer period. In a cross-sectional postal questionnaire study, widely used continuity of care indicators (such as COC, UPC) are not feasible [26]. The question used was understandable to Finns, and thus it gave us a good perspective of continuity among the population. Numerous other factors influencing continuity of care for example financial issues, physician availability and accessibility and facilities were not included to the study.

The independent factors were described by using measurements that are widely used in studies. The number of reported chronic diseases might be underestimated. The New York Heart Association classification and BDI questionnaire are easily applied and thus widely used, but there are limitations due to their subjective nature [34]. Some of the characteristics were continuous, and some of the information of the data was lost when transforming them binomially. Binary logistic regression was chosen to highlight the most important results. The data were collected 2012 but the results are still relevant. Continuity of care is more important factor in health care in Finland that it has been ever before.

Allocating a named GP to a patient indicates a promise of sustainability and a long-term doctor-patient relationship. Consequently, such a relationship reflects the continuity of care. In our study, the continuity of care indicated inter-personal care between the patient and the assigned and named GP. Patients feel safer when the same physician continues their treatment. To the patient, this is a matter of comprehensive understanding, not just quality of care [35]. Experiences of accessibility, presence, being listened to, and quality of care encourage the patient to contact the same physician [3]. Previous studies have shown the importance of a named GP in implementing continuity of care [36]. We confirmed the findings of previous studies: continuity of care markedly improves patient satisfaction [16, 22].

Finland has a history of patients being assigned a specific GP at the local health care centre, and this embraces the inter-personal continuity of care. Following new organizational methods, continuity of care has declined along with patient satisfaction. Our study emphasises importance of assigned and named GPs: specified GPs add satisfaction not only to service users, but to the whole population.

Higher patient satisfaction was most strongly associated with a lower BDI score. We found a difference in satisfaction when comparing respondents reporting depression to those with no reported depression. Depression declines with satisfaction figures, but still it is debatable whether the depressed participants of this study were less satisfied due to illness or worse health care services or treatment. Mental health problems lower satisfaction levels, and patients with severe anxiety disorder are less likely to be satisfied with treatment. With depressed patients, a patient-centred perspective increases satisfaction, but it does not influence the remission of depression [2, 13].

The gender of the patient and the physician did not influence patient satisfaction in other studies [8, 37], although there are findings of females being more satisfied [10]. In our study, males were marginally more often pleased with health care services. The difference in satisfaction between males and females was small but statistically significant because of the relatively large group sizes.

The large number of respondents in each group also explains the very small but statistically significant difference in satisfaction between higher and lower educated respondents. Less educated participants were more often satisfied with local health care services. This might be explained by their more frequent use of local health care services given the lack of occupational health or additional health care insurance.

In line with previous studies, elderly people were more satisfied with health care services compared to younger people, although contradictory results have also been found [7–9, 20]. Older people might consider their illness and treatments a burden [15], and dissatisfied elderly patients are more likely to be chronically ill [14]. Younger patients seem to appreciate shorter waiting times and longer appointments spent with the physician [1], and they are possibly more demanding than older patients [21]. Elderly people often have more chronic conditions, which could reduce satisfaction. Nevertheless, an association between older age and satisfaction was evident also in our study. Further studies are needed to find out possible differences in satisfaction between age cohorts.

Respondents with a disease common among Finns were significantly more often satisfied with local health care services than respondents without such a disease. The participants reported good experiences of the services when reporting high patient satisfaction. The difference between respondents with and without such a disease was clear. Healthier participants reported more often “I can’t say/I don’t know/I’m not sure” and were categorized as not satisfied. It is probable that the chronically ill have sought to have the same GP managing their care of the diseases demanding regular controls, thus affecting satisfaction. There was no difference in satisfaction when comparing participants with lung diseases to those without such diseases. The natures of the diseases are different, and in Finland for instance asthma or chronic bronchitis can be controlled by a nurse, who consults a GP only when necessary. Respondents with depression or another mental disease were less satisfied compared to those without such a disease, and this is one of our main results.

Although higher subjectively assessed quality of life is associated with greater service satisfaction [12], respondents with long-term illnesses were more often satisfied with health care services than those respondents without such diseases. In previous studies, patients rating their health status as good were more likely to be satisfied with the services [11], and also the chronically ill were more likely to be pleased with health care services [9, 10]. Several measurement tools and models have been created to estimate and produce effective treatment among the chronically ill [16–18]. The use of these models is associated with a higher quality of care and increasing the health of the chronically ill, although the use of models differs [18]. The disease does not decrease quality of life, which can also be a result of high-quality health care services. Experience of high-quality care improves patient satisfaction [16, 22]. The impact on health care service satisfaction was smaller with chronic diseases when compared to continuity of care. Participants with chronic diseases include part of the population that uses services regularly, and younger and healthier respondents might not have any experiences of the services.

In our data, the group with chronical diseases did not have a marked association with satisfaction in the multivariate analysis. Nevertheless, participants with chronic diseases were more often satisfied when compared to the healthier group. This could be explained by age and chronically ill patients’ tendency to search for continuous treatment.

The population was satisfied with local health care services. The percentages found in this survey were approximately the same as those found in previous studies in Finland [22], although the results are based on users’ opinions, not on the population. Two thirds of the population being satisfied gives us reason to ask whether this is adequate. Accessibility to public health care services has diminished in Finland and the reputation of the services is not always good. Still, the patients using the services are usually satisfied. Our study shows that chronic diseases in the population were not the reason for worse satisfaction figures. Satisfaction in Finland might have deteriorated due to a decrease in the continuity of care. The population with chronic conditions needs continuity of care to ensure good health care. Increasing continuity might also increase satisfaction with health care services.

## Conclusion

Patient satisfaction is widely used to define quality of care, and continuity of care is associated with satisfaction. In view of the changing working methods of GPs and the increasing numbers of chronically ill patients, continuity should be one of our top priorities. Treating patients with chronic diseases demands a comprehensive approach, and continuity of care can provide this. Depressed part of population is less satisfied with health care services. The treatment of depression should be considered when planning health care services. In pursuing greater patient satisfaction, we should increase continuity of care, as in addition to higher satisfaction figures, it brings multiple other benefits.

## Data Availability

All data analysed during this study are included in this published article [and its supplementary information files].

## Abbreviations

BDI: Beck’s Depression Inventory
BMI: Body Mass Index
CI: Confidence Interval
COC: Continuity of Care Index
GP: General Practitioner
HeSSup: Health and Social Support study
NICE: National Institute for Health and Care Excellence
NYHA: New York Heart Association
OR: Odds Ratio
SPSS: IBM SPSS Statistics program
TIA: transient ischemic attack
UPC: Usual Provider Continuity Index
